# Alternative Splicing of RIOK3 Engages the Noncanonical NFκB Pathway during Rift Valley Fever Virus Infection

**DOI:** 10.3390/v15071566

**Published:** 2023-07-18

**Authors:** Thomas Charles Bisom, Hope Smelser, Jean-Marc Lanchy, J. Stephen Lodmell

**Affiliations:** 1 Department of Chemistry and Biochemistry, University of Montana, Missoula, MT 59801, USA; thomas.bisom@umconnect.umt.edu (T.C.B.); hope.smelser@umconnect.umt.edu (H.S.); 2 Division of Biological Sciences, University of Montana, Missoula, MT 59801, USA; jean-marc.lanchy@umontana.edu; 3 Center for Biomolecular Structure and Dynamics, University of Montana, Missoula, MT 59801, USA

**Keywords:** noncanonical NFκB pathway, Rift Valley fever virus, RIOK3, innate immunity, alternative splicing

## Abstract

Although the noncanonical NFκB pathway was originally identified as a cellular pathway contributing to lymphoid organogenesis, in the past 20 years, its involvement in innate immunity has become more appreciated. In particular, the noncanonical NFκB pathway has been found to be activated and even exploited by some RNA viruses during infection. Intriguingly, activation of this pathway has been shown to have a role in disrupting transcription of type 1 interferon (IFN), suggesting a rationale for why this response could be co-opted by some viruses. Rift Valley fever virus (RVFV) is a trisegmented ambisense RNA virus that poses a considerable threat to domestic livestock and human health. Previously, we showed the atypical kinase RIOK3 is important for mounting an IFN response to RVFV infection of human epithelial cells, and shortly following infection with RVFV (MP12 strain), RIOK3 mRNA is alternatively spliced to its X2 isoform that encodes a truncated RIOK3 protein. Alternative splicing of RIOK3 mRNA has an inhibitory effect on the IFN response but also stimulates an NFκB-mediated inflammatory response. Here, we demonstrate alternative splicing of RIOK3 mRNA is associated with activation of the noncanonical NFκB pathway and suggest this pathway is co-opted by RVFV (MP12) to enhance viral success during infection.

## 1. Introduction

Careful co-ordination of interferon (IFN) and inflammatory responses to viral infection is essential for robust cellular immunity as well as avoidance of collateral tissue damage caused by overblown immune responses. Viruses have evolved many strategies to dysregulate discrete pathways to evade cellular responses to infection. Such dysregulation can be harmful to the host. For example, with the recent COVID-19 pandemic, exacerbated inflammatory responses coupled with a muted IFN response were frequently observed in the most severe clinical cases [1,2]. Therefore, understanding how viruses exploit innate immune pathways within the cell could be valuable for developing countermeasures to severe viral infections.

Rift Valley fever virus (RVFV; order *Bunyavirales*, family *Phenuiviridae*, genus *Phlebovirus* [3]) is an arthropod-borne RNA virus that poses a considerable threat to human health and agriculture. Transmission typically occurs from mosquitoes of the *Aedes* and *Culex* genera to humans and livestock, such as sheep, goats, and cattle [4,5,6]. Human infections can also occur by handling meat, blood, or milk or inhaling aerosolized droplets from fluids of infected animals [7]. Symptoms of human infection range from flu-like symptoms to hemorrhagic fever [8,9]. Hepatic complications, renal failure, and miscarriage have also been reported in humans [10,11], and these pathologies have likewise been observed in animal models of Rift Valley fever and in livestock [12,13,14,15,16]. Due to its virulence and risk of being transmitted through aerosolization, RVFV is also considered a threat to national security in some countries, including the United States, where it is considered a USDA/HHS Overlap Select Agent [17,18]. Understanding the host innate immunologic responses to RVFV infection is, therefore, valuable to global human health and security.

RVFV has an ambisense tripartite RNA genome and, following infection of a cell, genomic viral RNA is detected predominantly by the retinoic acid inducible gene I (RIG-I) intracellular receptor [19,20]. After detecting viral RNA, RIG-I associates with mitochondrial antiviral signaling protein (MAVS) and, following recruitment of TANK binding kinase 1 (TBK1) to MAVS and subsequent phosphorylation of interferon regulatory factor 3 (IRF3) by TBK1, a type 1 IFN response is initiated [21,22,23]. This results in transcription and translation of type 1 IFNs, such as IFNβ, which, following translation, activate the JAK/STAT pathway through autocrine and paracrine signaling [24,25]. This pathway then results in transcription of a subset of genes, the IFN stimulated genes, that induce an antiviral state in the cell [26,27,28].

**Figure 1:**
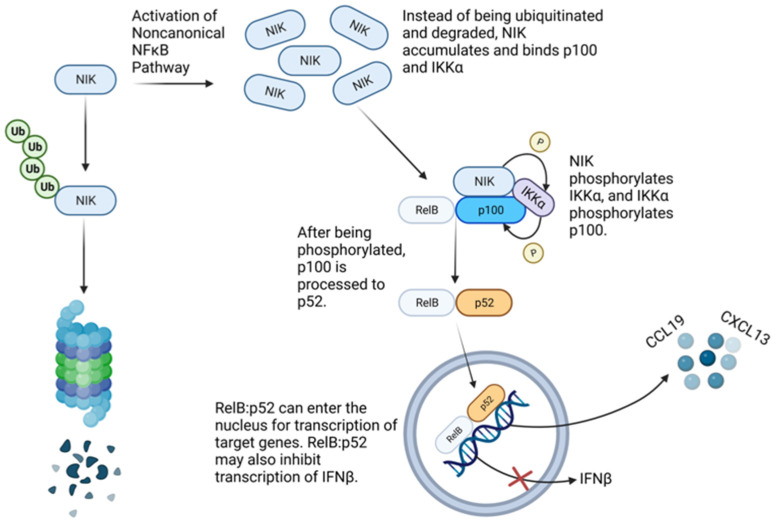
Noncanonical NFκB pathway overview. See text for details. Typically, the onset of the noncanonical NFκB pathway is delayed compared to that of the canonical NFκB pathway. In this study, however, we show engagement of the noncanonical pathway prior to or concomitant with the canonical pathway during RVFV MP12 infection.

Although the noncanonical NFκB pathway (also known as the alternative NFκB pathway) was originally identified as a cellular pathway involved in adaptive immunity and secondary lymphoid organogenesis [29,30,31,32], it has more recently become appreciated in cellular innate immunity for its role in transcription of inflammatory cytokines and chemokines during viral infection [33,34,35,36,37]. Intriguingly, it has also been shown to disrupt transcription of type 1 IFN [38]. During activation of the noncanonical NFκB pathway, an activating signal frees NFκB-inducing kinase (NIK; also known as MAP3K14) from ubiquitination by an E3 ubiquitin ligase complex comprised of TRAF3 (linking NIK to the complex), TRAF2, and cIAP1/2 and thereby spares NIK from proteasomal degradation [39,40,41,42,43]. NIK then phosphorylates IKKα [44]. Phosphorylated IKKα then phosphorylates p100 (also known as NFκB2) dimerized with RelB in the cytosol, leading to proteolytic processing of p100 to p52 [33,45,46,47]. The noncanonical NFκB dimer of p52 complexed with RelB then enters the nucleus for transcription of genes specific to the noncanonical NFκB pathway, such as CXCL13 (also knowns as BLC) and CCL19 (also known as ELC) [48]. Furthermore, while in the nucleus, the noncanonical NFκB dimer is thought to inhibit the IFN response in part by competing with the canonical (also known as classical) NFκB dimer for the κB site at the IFNβ promoter (Figure 1) [38].

Canonical (p50:RelA, p50:c-Rel, and p50:p50) and noncanonical (p52:RelB) NFκB dimers belong to the same family of NFκB transcription factors and share a Rel homology domain [49,50,51,52], but there are key differences in their transcriptional activities and in how and when these transcription factors become active [53,54,55]. For example, in order for canonical NFκB dimers to become transcriptionally active, they must be freed from members of inhibitor of κB (IκB) family of inhibitory proteins in the cytosol, such as IκBα [56,57,58]. Upon phosphorylation, IκB proteins are ubiquitinated and then degraded by the proteosome, allowing canonical NFκB dimers to enter the nucleus for transcription of target genes [59,60,61].

There are several other characteristic differences between the canonical and noncanonical NFκB pathways. Typically, activation of the canonical pathway occurs almost immediately upon immune stimulation, but the noncanonical pathway has a much slower onset and depends on de novo protein synthesis [32,33,62,63]. Distinct receptors activate the noncanonical and canonical pathways, and activation of the noncanonical pathway is more selective and exclusive to a subset of receptors [32,33]. The noncanonical pathway can be activated through binding of the LTβR receptor with ligands, such as LIGHT [64,65]. Activation of the canonical pathway is typified by binding of TNFα with TNFR1 receptor [66]. However, both canonical and noncanonical pathways are known to be interwoven, and activation of one pathway can sometimes result in or even rely on activation of the other. For example, when the noncanonical NFκB pathway was first described, expression (but not processing) of p100 was shown to depend on the canonical NFκB dimer, p50:RelA [65].

In addition to a type 1 IFN response that is initiated by RIG-I detection of viral RNA, both canonical and noncanonical NFκB pathways can also be activated when RIG-I associates with MAVS [36,67]. We and others have shown the atypical kinase RIOK3 is important for regulating a RIG-I-induced IFN response [68,69,70,71,72]. However, shortly following RVFV infection, a shift toward alternative splicing of RIOK3 mRNA to its X2 isoform occurs, culminating in a diminished IFN response but an elevated NFκB-mediated inflammatory response in human epithelial cells [70,73]. Therefore, promoting this alternative splicing of RIOK3 mRNA could serve as a means for RVFV to evade a type 1 IFN response and potentially lead to a dysregulated innate immune response. Here, we show the noncanonical NFκB pathway is activated early during RVFV (MP12 strain) infection and that alternative splicing of RIOK3 occurs specifically during activation of the noncanonical NFκB pathway. Furthermore, the alternatively spliced isoform of RIOK3, called RIOK3 X2, aids in expression of p100, which diminishes the type 1 IFN response. Collectively, these observations suggest that RVFV infection and alternative splicing of RIOK3 mRNA accompany induction of the noncanonical NFκB pathway, which decreases IFN production and enhances viral multiplication.

## 2. Materials and Methods

### 2.1. Cell Culture, Viruses, and Infections

Vero 76, human embryonic kidney 293 (HEK 293), and hepatocarcinoma (HepG2) cells were obtained from ATCC (Manassas, VA, USA). Vero 76 and HEK 293 cells were cultured in Dulbecco’s Modified Eagle Medium (DMEM) containing 10% fetal bovine serum (FBS) and penicillin + streptomycin (pen/strep) (Thermo Fisher Scientific, Waltham, MA, USA), and HepG2 cells were grown in 1:1 DMEM:F12 containing 10% FBS and pen/strep (Thermo Fisher Scientific). Since renal failure and hepatic complications have frequently been observed in severe cases of RVFV infection, HEK 293 and HepG2 cells were selected for use in this study. Experiments were initiated when cells were near confluency (70–90%). Furthermore, the attenuated biosafety level 2 (BSL2) laboratory RVFV strain MP12 (kindly provided by Brian Gowen Utah State University, Logan, UT, USA) was used for all viral infections. MP12 is an attenuated RVFV strain derived from the wt ZH548 isolate, and this strain was used because it can be handled in BSL2 containment. RVFV MP12 harbors mutations in all three of its genomic segments to mitigate its pathogenicity [74], but many of the virological attributes of MP12, such as its cytopathogenicity in cell culture, are still shared with RVFV ZH548, making MP12 a good surrogate for initial studies of RVFV. MP12 viral stocks were propagated in Vero 76 cells, and the second parental generation was used for infections in experiments. During experimental infections, cells were incubated with virus in DMEM or DMEM:F12 containing no FBS and no pen/strep for 1 h. Media were then exchanged with DMEM or DMEM:F12 containing 2% FBS and pen/strep, and the cells were incubated at 37 °C, 5% CO_2_; see text for specific times when cells were harvested post-infection. Quantification of infectious dose was performed in TCID50 assays described by Smith et al. [75] with Vero 76 cells, and TCID50/mL values were obtained using the Reed and Muench Method. Manipulations of the viruses used in this study are compliant with both the Institutional Biosafety Committee at the University of Montana, Missoula, and NIH requirements in regard to their handling under BSL2 containment conditions.

### 2.2. Reagents

TNFα and LIGHT were purchased from BioLegend (San Diego, CA, USA). Prior to treating cells with TNFα or LIGHT, growth media were removed; cells were washed with PBS, and PBS was exchanged with fresh DMEM media containing 10 or 2% FBS and pen/strep. Then, when treating with TNFα, TNFα was added such that its final concentration in each well was 20 ng/mL, and when treating with LIGHT, LIGHT was added to each well to reach a final concentration of 50 ng/mL. After treating with either reagent, cells were then incubated at 37 °C, 5% CO_2_; see text for incubation times used in each assay.

### 2.3. Plasmids, Oligonucleotides, and Transfections

Plasmid transfections were accomplished with Lipofectamine 2000 (Thermo Fisher Scientific). In these transfections, growth media were removed, cells were washed with PBS, and PBS in each well was exchanged with a volume of OptiMEM transfection media equal to half the volume of growth media. The cells were then incubated at 37 °C, 5% CO_2_. While the cells were incubating, transfection master mixes were prepared. When preparing Lipofectamine master mixes, Lipofectamine was added to OptiMEM media and incubated at room temperature for 5–20 min. Lipofectamine was added to each master mix such that the ratio of μL Lipofectamine 2000 to μg of construct transfected would be ~2.5 per well. After incubation of Lipofectamine master mixes, these mixtures were added to DNA construct master mixes comprising construct DNA and OptiMEM, and the resulting transfection mixture was left to incubate at room temperature for 20 min to allow assembly of DNA: liposome complexes. Transfection mixtures were then dispensed into wells with cells incubated in OptiMEM media, and the mixtures were added to each well at a volume equal to half the volume of growth media. Before any downstream treatments were initiated, cells were incubated with the transfection mixtures at 37 °C, 5% CO_2_ for at least 24 h to allow sufficient expression of the constructs. The RIOK3 X2 construct, which we previously showed expressed the predicted protein/peptide, was expressed in a phRL-CMV backbone, and the empty vector (EV) control plasmid described in the text was phRL-CMV backbone lacking any insert [70].

### 2.4. RNA Extractions and Reverse Transcription

When extracting RNA, cells were lysed with TRIzol (Thermo Fisher Scientific), and RNA was extracted from the reagent according to the manufacturer’s protocol. To separate RNA into an aqueous layer, lysates were first treated with 0.2 mL chloroform per 1 mL TRIzol. Following vortex and incubation at room temperature for 10 min, the lysates were spun at 12,000× *g* and 4 °C for 15 min. Aqueous layers containing RNA were then transferred to new tubes, and to precipitate the RNA, 2.5 μL of 4 mg/mL glycogen and 0.5 mL isopropanol per 1 mL of TRIzol used were added. After vortex and incubation at room temperature for 10 min, RNA samples were then spun at 12,000× *g* and 4 °C for 10 min. Supernatant was then removed, and the RNA pellets were washed with 1 mL of 75% ethanol per 1 mL of TRIzol used. Washes were accomplished by brief vortex of the pellets in 75% ethanol and centrifugation at 7600× *g* and 4 °C for 5 min. The 75% ethanol solution was then removed, and the pellets dried at room temperature for 5–10 min. RNA was then resuspended in nanopure H_2_O, incubated at 55–60 °C for 10–15 min, and stored at −80 °C or used immediately. Extracted RNA was then reverse transcribed to cDNA with Maxima H Minus Reverse Transcriptase (Thermo Fisher Scientific). Prior to reverse transcription, RNA in each sample was diluted to ~100 ng/μL. Diluted RNA was incubated with random hexamer primers and dNTPs at 65 °C for 5 min, chilled, and reverse transcription reagents (reverse transcription buffer and Maxima H Minus Reverse transcriptase) were then added. After addition of reverse transcription reagents, samples were incubated at 25 °C for 10 min., 50 °C for 30 min, and 85 °C for 5 min. Resulting cDNA was then either immediately used or stored at −20 °C.

### 2.5. RT-qPCR

RT-qPCR measurements were obtained using SYBR Green Master Mix (Thermo Fisher Scientific) and the CFX Connect Real-Time PCR Detection System (Bio-Rad, Hercules, CA, USA). First, cDNA was diluted 1:5 in nanopure H_2_O. Measurements were obtained using a 384-well plate, and each well with sample contained 5 μL SYBR Green Master Mix, 1 μL forward + reverse 10 μM primer mix, and 4 μL diluted cDNA. cDNA was then amplified and measured under the following conditions: (1) 50 °C for 2 min, (2) 95 °C for 2 min, (3) 95 °C for 15 s, (4) 60 °C for 1 min, (5) plate read, (6) go to (3) 39×, (7) 65 °C for 31 s, (8) 65 °C for 5 s + 0.5 °C/cycle Ramp 0.5 °C/s, (9) plate read, (10) go to 8) 60×. Relative normalized expression was quantified in CFX Maestro (Bio-Rad) using the ∆∆Ct method, and RNA was normalized to GAPDH (see Table S1 for qPCR primer sequences).

### 2.6. Gel Electrophoresis

Prior to electrophoresis, cDNA was amplified by PCR using InPhusion Flash Hi-Fidelity PCR Master Mix (Thermo Fisher Scientific). Each reaction mixture consisted of 10 μL Phusion Flash PCR Master Mix, 1 μL of 10μM forward primer, 1 μL of 10 μM reverse primer, 6 μL nanopure H_2_O, and 2 μL cDNA. cDNA was then amplified under the following conditions: (1) 98 °C for 10 s, (2) 98 °C for 1 s, (3) 65 °C for 5 s, (4) 72 °C for 15 s, (5) go to (2) 29×, (6) 72 °C for 1 min, (7) 4 °C forever. Amplified cDNAs were then resolved on a 1% agarose gel at 80 V. After rocking gels for 10 min in a 1× TAE bath containing ethidium bromide, images were obtained using a Gel Doc XR + Molecular Imager (Bio-Rad). Figures show representative samples of biological duplicates or triplicates.

### 2.7. Western Blotting 

Cells harvested for Western blotting were grown in 6-well plates. Prior to harvest, media was removed, and the cells were washed with PBS (Thermo Fisher Scientific). To remove cells from the plate, cell scraping or trypsin (Thermo Fisher Scientific) was used. When scraping cells, after PBS wash, 5 mL PBS were added per well while the 6-well plate was on ice. Cells were scraped, and suspended cells were transferred to conical centrifuge tubes kept on ice. A total of 5 mL of PBS were again added per well, and scraping and transfer of suspensions to centrifuge tubes was repeated. The cells were then centrifuged at ~1000 rpm, 4 °C for 5–10 min. PBS was then removed, and the cells were either lysed immediately or stored at −80 °C until lysis. When removing cells from 6-well plates with trypsin, 1 mL trypsin was added per well after washing with PBS. Cells were incubated at 37 °C, 5% CO_2_ for 2–5 min until they no longer adhered to the plate(s), and the suspensions were then transferred to conical centrifuge tubes and were centrifuged at ~1000 rpm, 4 °C for ~5 min. Trypsin was removed, and cell pellets were resuspended in chilled PBS (10 mL/cell pellet). Resuspensions were centrifuged at ~1000 rpm, 4 °C for 5–10 min, and PBS was removed. Cells were either lysed immediately or stored at −80 °C until lysis. Cells were lysed with radioimmunoprecipitation buffer (RIPA; 10 mM Tris HCl (pH 8.0), 1 mM EDTA, 0.5 mM EGTA, 1% Triton X-100, 0.1% sodium deoxycholate, 0.1% SDS, 140 mM NaCl) containing Halt Protease Inhibitor Cocktail (Thermo Fisher Scientific). 

Lysates were either used immediately or stored at −80 °C. Total protein concentrations in lysates were then measured with a BCA assay (Thermo Fisher Scientific) and diluted accordingly with RIPA to reach as equal concentrations as possible. Proteins were then resolved by denaturing polyacrylamide gel electrophoresis using Tris-Glycine SDS Running Buffer (25 mM Tris Base, 192 mM Glycine, 0.1% SDS) and 8% acrylamide Tris-Glycine precast gels (NuSep, Germantown, MD, USA). Proteins were transferred from the gel to PVDF membrane (MilliporeSigma, Burlington, MA, USA) at 30 V for 960 min in transfer buffer (25 mM Tris base, 0.19 M glycine, 20% methanol). Membranes were blocked with non-fat milk (5% *w/v* non-fat dry milk in TBST) at room temperature for 1 h and subsequently washed with Tris-buffered saline Tween-20 (TBST; 20 mM Tris-HCl pH 7.5, 0.15 M NaCl, 0.1% Tween-20) 3 times, 5 min each time. Incubation in 1° antibodies was then performed at room temperature for 2 h or 4 °C overnight. TBST washes were again performed 3 times, 5 min each time, and the membrane was incubated with horseradish peroxidase (HRP) 2° antibodies at room temperature for 1 h. After washing with TBST 3 times, 10 min each time, membranes were incubated in visualization solution (Solution A: 100 mM Tris-HCl pH 8.5, 2.5 mM luminol, 0.396 mM coumaric acid + Solution B: 100 mM Tris-HCl pH 8.5, 0.0192% H_2_O_2_) and visualized on an Image Reader LAS-3000 (FujiFilm, Greenwood, SC, USA). When visualizing proteins specific to more than one 1° antibody, the membrane was severed at a molecular weight distinguishing the two proteins and incubated separately with distinct 1° antibodies. The 1° antibodies used were rat anti-p100/p52, mouse anti-IκBα, mouse anti-β-tubulin, and mouse anti-GAPDH and were all purchased from BioLegend. The 2° antibodies used were HRP goat anti-rat IgG (BioLegend) and HRP goat anti-mouse IgG (Sigma-Aldrich, St. Louis, MO, USA). Where applicable, band intensities were quantified by ImageJ software [76]. See Table S2 for ratios of antibody/blocking solution used. Figures show representative samples of biological duplicates. 

### 2.8. Statistical Analysis

All experiments were replicated in biological duplicate or triplicate. Western blots shown are representative examples of duplicated or triplicated experiments. Statistically significant differences in the quantitative RT-PCR data were determined using a two-tailed unpaired Student’s *t*-test in GraphPad Prism 8. Error bars are reported as standard error of the mean

## 3. Results

### 3.1. The Noncanonical NFκB Pathway Is Activated Early in RVFV (MP12) Infection

As described above, alternative splicing of RIOK3 mRNA is observed upon RVFV infection. Moreover, the suppression of IFN expression and enhanced expression of a subset of inflammatory cytokines is observed when alternatively spliced RIOK3 mRNA is expressed [70,73]. Suppression of IFN and enhanced expression of certain inflammatory cytokines are also characteristics of activation of the NFκB noncanonical pathway [33]. Therefore, we sought to determine whether the noncanonical NFκB pathway is activated during RVFV infection and whether alternative splicing of RIOK3 mRNA has a role in this pathway. First, to specifically determine whether infection with RVFV (strain MP12) activated the noncanonical NFκB pathway, we measured expression of genes that are indicative of this pathway.

**Figure 2:**
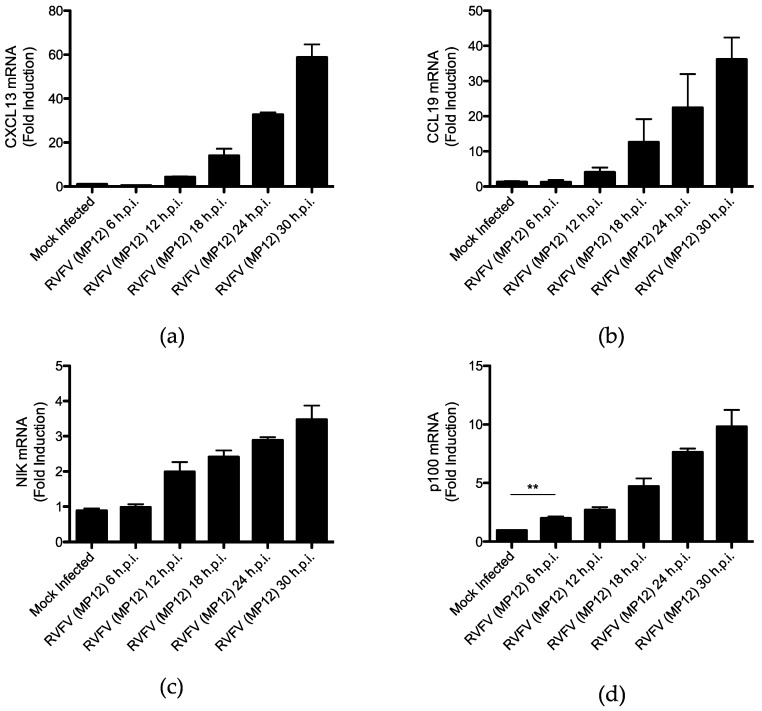
The noncanonical NFκB pathway is activated during RVFV MP12 infection. RVFV-infected cells were lysed at the times post-infection indicated, and mRNAs of the noncanonical NFκB pathway genes (**a**) CXCL13, (**b**) CCL19, (**c**) NIK, and (**d**) p100 were quantified by RT-qPCR, normalized to GAPDH mRNA levels. Plots are representative of the data as the mean value of 3 biological replicates +/− SEM. Student’s *t*-test: ** *p* < 0.01.

Since CXCL13 and CCL19 have been shown to be transcribed specifically during activation of the noncanonical NFκB pathway [48], mRNAs of these chemokines were measured at multiple timepoints following RVFV MP12 infection. HEK 293 cells were infected with MP12 at an MOI of 2, and cells were harvested at 6, 12, 18, 24, and 30 h post-infection (h.p.i.). CXCL13 and CCL19 mRNAs at these times were then measured by RT-qPCR, and throughout RVFV MP12 infection, relative expression (normalized to GAPDH) of these chemokines continued to increase, suggesting progressive activation of the noncanonical NFκB pathway (Figure 2a,b). Intriguingly, relative expression of NIK and p100 also increased throughout infection, and importantly, there was a statistically significant increase in p100 expression as early as 6 h.p.i. (Figure 2c,d). The same increase in relative expression of CXCL13, CCL19, NIK, and p100 was not observed in mock infected cells harvested at the same time points, and relative expression of CXCL13, CCL19, NIK, and p100 did not change significantly from 0 to 30 h in these cells (Figure S1). Furthermore, in HepG2 cells, relative normalized expression of CXCL13, NIK, and p100 also increased significantly in RVFV-MP12-infected cells, and although the difference in relative expression of CCL19 between infected and mock infected cells did not rise to statistical significance, CCL19 expression was reproducibly elevated in RVFV-MP12-infected cells (Figure S2).

**Figure 3:**
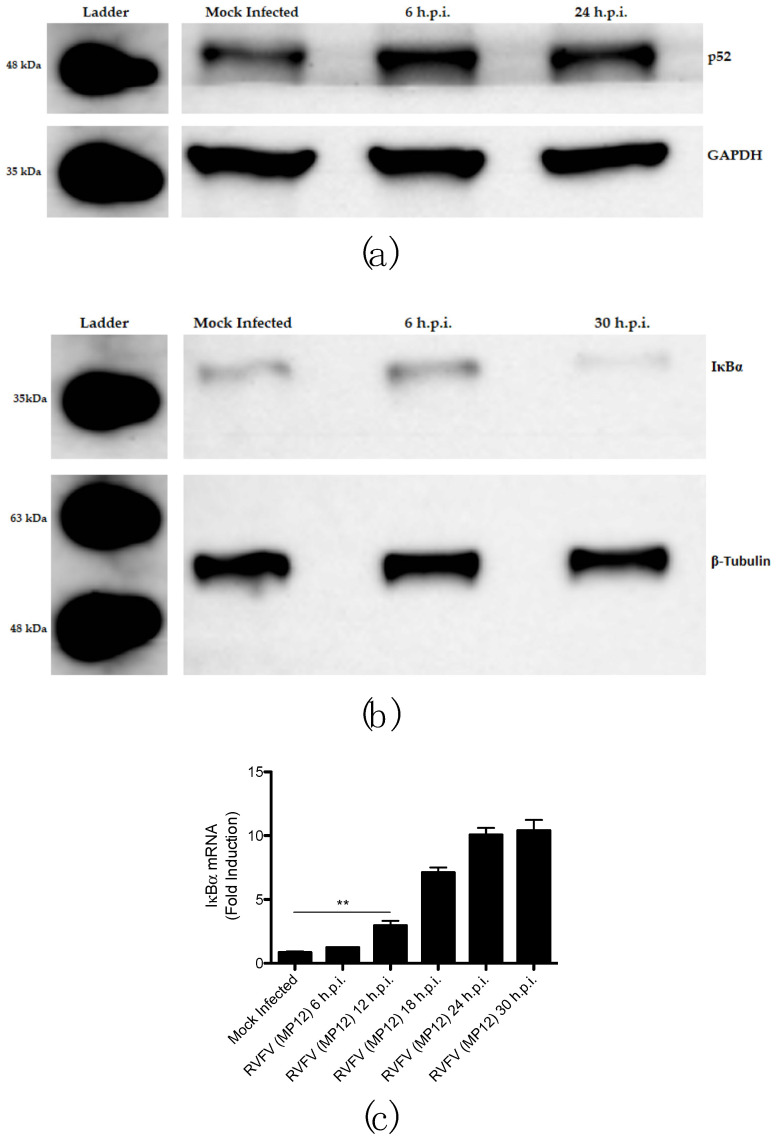
Activation of the noncanonical NFκB precedes activation of the canonical NFκB pathway during RVFV MP12 infection. Activation of the noncanonical pathway was assessed by (**a**) measuring p100 processing, and activation of the canonical pathway was assessed by (**b**) measuring degradation of IκBα via Western blotting. Activation of the canonical pathway was also measured, via RT-qPCR, by (**c**) assessing relative mRNA expression of IκBα normalized to GAPDH. Images in (**a**,**b**) are representative samples of biological duplicates. Furthermore, p52 levels were also assessed by Western blotting with the same samples shown in (**b**), and quantification of band intensities is shown in Figure S3b. In these samples, p52 is increased at 6 h.p.i. and is further increased at 30 h.p.i. The plot shown in (**c**) presents the data as the mean value of 3 biological replicates +/− SEM. Student’s *t*-test: ** *p* < 0.01.

Processing of p100 to p52 is a key step in activation of the noncanonical NFκB pathway. p100 inhibits RelB from translocating to the nucleus and thereby inhibits transcription of noncanonical NFκB genes, but when p100 is processed to p52 the RelB/p52 dimer is favored to enter the nucleus for transcription of noncanonical NFκB genes [32,77]. Notably, constitutive processing of p100 in most cell types is lacking, thereby limiting and strictly regulating activation of the noncanonical NFκB pathway. For example, in most cells, p100 predominates largely over p52, and when p100 is overexpressed it is scarcely converted to p52. Therefore, any noticeable increase in p52 protein levels is strongly indicative of activation of the noncanonical NFκB pathway [47,78]. To assess p100 processing, protein levels of p52 were measured during RVFV MP12 infection by Western blotting. HEK 293 cells were infected with MP12 for 6 and 24 h, and p52 levels were then visualized using an antibody that recognizes both p100 and its proteolytic product, p52. Levels of p52 appear to increase as early 6 h.p.i. and remain increased at 24 h.p.i., suggesting processing of p100 to p52 indeed occurs early following RVFV MP12 infection (Figure 3a). Quantification of band intensity further supports these results (Figure S3a).

Activation of the canonical NFκB pathway during RVFV MP12 infection was also assessed by measuring protein levels of IκBα during infection. Since IκBα protein inhibits transcriptional activity of canonical NFκB dimers, IκBα is expected to decrease with activation of the canonical pathway. Therefore, similar to the appearance of p52 being a hallmark of activation of the noncanonical pathway, decreased IκBα is indicative of activation of the canonical pathway [59,60,61]. IκBα protein level was probed via Western blotting at 6 and 30 h.p.i., and intriguingly, in MP12-infected HEK 293 cells, a decrease in IκBα protein is not observed at 6 h.p.i. but is at 30 h.p.i., suggesting the canonical NFκB pathway is activated after the noncanonical pathway (Figure 3b). Normally, induction of the noncanonical pathway is delayed and activated after the canonical pathway [32,33]. Early activation of the noncanonical pathway and delayed onset of the canonical pathway was also observed in RVFV-MP12-infected HepG2 cells, in which degradation of IκBα was not observed at 6 h.p.i., but accumulation of p52 was observed (Figure S4).

Although IκBα protein level decreases with activation of the canonical NFκB pathway, IκBα mRNA has been shown to increase with activation of the canonical pathway, thereby contributing to the oscillatory nature of this pathway [79]. Therefore, IκBα mRNA was also measured by RT-qPCR throughout infection of HEK 293 cells with MP12, and it did not increase considerably above baseline until 12 h.p.i. (Figure 3c). Since p100 mRNA expression is significantly elevated by 6 h.p.i. and p52 was also shown to appear by this time, yet IκBα mRNA was not significantly elevated until 12 h.p.i., these data show that p100 processing (and thereby activation of the noncanonical pathway) precedes activation of the canonical pathway during RVFV MP12 infection. Collectively, these results suggest the noncanonical NFκB pathway is activated early in RVFV MP12 infection and even before the canonical NFκB pathway, implying the noncanonical pathway, and specifically p100 processing, may be co-opted by RVFV as a means to subvert the antiviral IFN response.

### 3.2. Alternative Splicing of RIOK3 Correlates with Activation of the Noncanonical NFκB Pathway

Our laboratory has shown alternative splicing of the atypical kinase RIOK3 occurs throughout RVFV infection [70,80]. Furthermore, alternative splicing of RIOK3 per se results in a diminished IFN response and elevated NFκB-mediated inflammatory response in epithelial cells [73], which are also characteristics of activation of the noncanonical NFκB pathway. It is notable that expression of the RVFV virulence factor NSs inhibits expression of IFN [81,82]. However, the isoform of RIOK3 expressed from the alternatively spliced mRNA can itself inhibit expression of IFN in the absence of NSs [73]. Furthermore, as shown above, activation of the noncanonical pathway occurs early in RVFV MP12 infection. Therefore, we sought to determine whether alternative splicing of RIOK3 is a programmed event during general activation of the noncanonical NFκB pathway.

**Figure 4:**
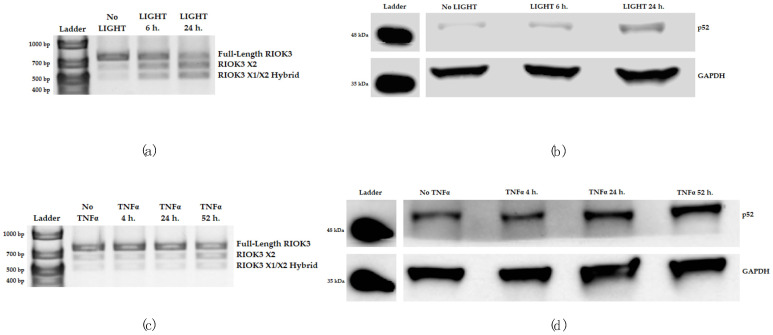
Alternative splicing of RIOK3 occurs specifically during activation of the noncanonical NFκB pathway. At 0, 6, and 24 h post-LIGHT treatment, (**a**) alternative splicing of RIOK3 and (**b**) activation of the noncanonical pathway were assessed. RIOK3 alternative splicing was assessed by RT-PCR and gel electrophoresis, and activation of the noncanonical pathway was shown through probing for p52 via Western blotting. At 0, 4, 24, and 52 h post-TNFα treatment, (**c**) alternative splicing of RIOK3 and (**b**) activation of the noncanonical pathway were also assessed in the same manner as in (**a**,**b**). Images in (**a**–**d**) show representative samples of biological duplicates or triplicates.

To assess alternative splicing of RIOK3 as a function of activation of the noncanonical NFκB pathway, cDNA of RIOK3 mRNA was amplified by RT-PCR between exons 5 and 10, allowing up to three bands to be visualized in an agarose gel (Figure 4). The slowest migrating band corresponds to full-length canonically spliced RIOK3 mRNA; the middle band corresponds to mRNA of RIOK3′s principal alternatively spliced isoform, RIOK3 X2, which lacks part of exon 8, and the fastest migrating band corresponds to mRNA of a RIOK3 X1/X2 hybrid that lacks all of exon 7 and part of exon 8. Notably, the alternatively spliced mRNAs contain a premature stop codon that would yield a truncated protein after translation.

To examine the kinetics of RIOK3 mRNA splicing upon NFκB pathway activation, HEK 293 cells were treated with the noncanonical NFκB pathway agonist LIGHT for 6 and 24 h, and alternative splicing of RIOK3 was assessed as mentioned above. With LIGHT treatment, alternative splicing of RIOK3 was observed by 6 h and appeared to increase up to 24 h after LIGHT treatment (Figure 4a). This correlated with p100 processing, as indicated by an increase in p52 levels (Figure 4b). However, when treating HEK 293 cells with TNFα, which immediately activates the canonical NFκB pathway, alternative splicing of RIOK3 was not observed between 4 and 24 h post-TNFα treatment, suggesting alternative splicing is not programmed into activation of the canonical NFκB pathway (Figure S5). 

Since the noncanonical NFκB pathway is known to also become activated following TNFα treatment but normally has delayed kinetics compared to the canonical pathway [32,33], we also assessed alternative splicing of RIOK3 at later times post-TNFα treatment. By 52 h post-TNFα treatment, alternative splicing of RIOK3 was observed (Figure 4c). The kinetics of appearance of RIOK3 alternative splicing corresponded with increased p52 levels, which appear to begin to increase at 24 h and become abundant by 52 h post-TNFα treatment (Figure 4d). These results suggest alternative splicing of RIOK3 might occur upon activation of the noncanonical NFκB pathway but not during activation of the canonical NFκB pathway.

**Figure 5:**
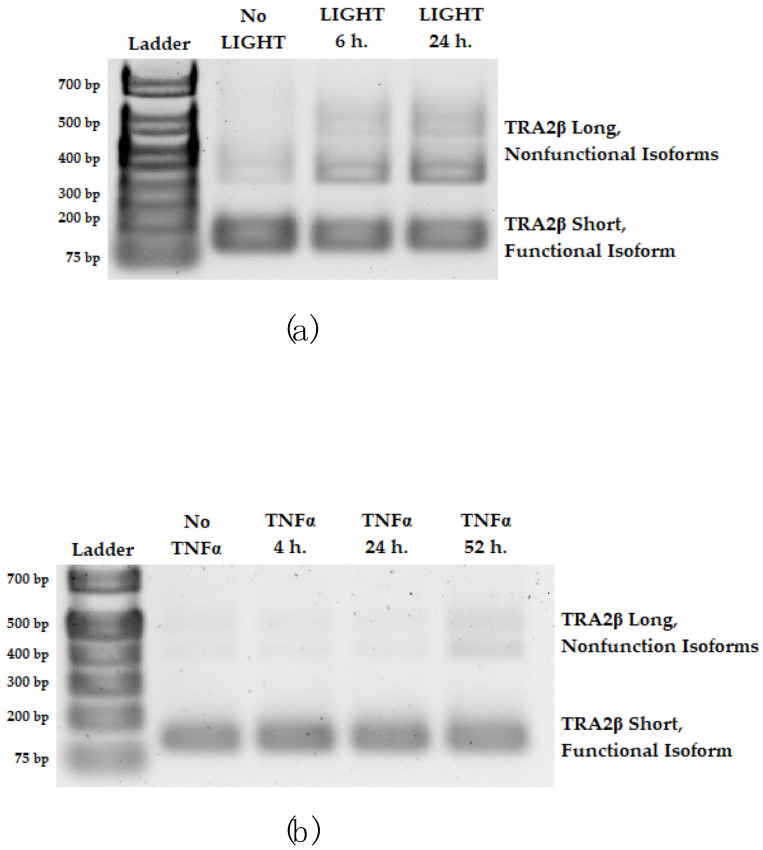
Alternative splicing of TRA2β to include a poison exon and produce nonfunctional isoforms also occurs specifically during activation of the noncanonical NFκB pathway. Alternative splicing of TRA2β was assessed during (**a**) LIGHT and (**b**) TNFα treatment using RT-PCR and gel electrophoresis. Images in (**a**,**b**) show representative samples of biological duplicates or triplicate.

Our laboratory recently showed that the splicing factor TRA2β plays an important role in RIOK3 splicing patterns. In fact, RIOK3 expression levels are governed by alternative splicing of TRA2β mRNA. TRA2β was shown to be important for canonical splicing of RIOK3 mRNA to full-length RIOK3 [80]. However, when TRA2β is alternatively spliced to incorporate a poison exon, nonfunctional mRNA isoforms of TRA2β with higher molecular weights are produced and rapidly degraded by nonsense-mediated decay [83,84]. Reduction in TRA2β was shown to result in alternative splicing of RIOK3 to RIOK3 X2 [80]. Therefore, TRA2β mRNA processing was also probed during LIGHT or TNFα treatment. During LIGHT treatment, TRA2β nonfunctional isoforms were observed 6 and 24 h post-treatment. During TNFα treatment, TRA2β nonfunctional isoforms are also observed by 52 h post-treatment (Figure 5a,b). These results corroborate our finding that alternative splicing of RIOK3 may in fact be part of the noncanonical NFκB response.

### 3.3. Expression of RIOK3 X2 during RVFV (MP12) Infection Increases p100 Expression and Decreases IFNβ Expression

Since alternative splicing of RIOK3 occurs during RVFV infection and the splicing shift appears concurrently with activation of the noncanonical NFκB pathway, we then investigated whether RIOK3′s alternatively spliced isoform, RIOK3 X2, has a direct effect on innate immune responses during RVFV MP12 infection.

**Figure 6:**
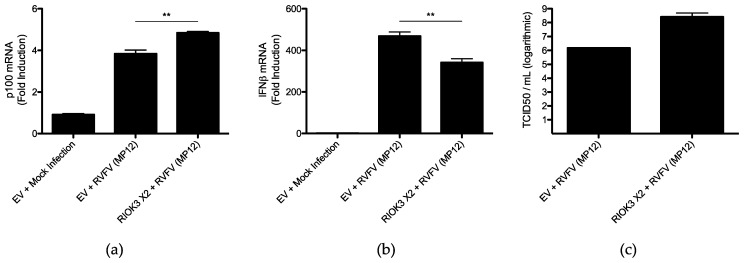
Overexpression of RIOK3 X2 in RVFV-MP12-infected cells results in increased p100 expression, decreased IFNβ expression, and increased RVFV MP12 TCID50/mL. Relative expression of (**a**) p100 and (**b**) IFNβ were measured by RT-qPCR, normalizing to GAPDH mRNA. TCID50/mL values were obtained from TCID50 assays using Vero 76 cells. In (**a**–**c**), HEK 293 cells were transfected with EV control plasmid or RIOK3 X2 for 24 h and subsequently infected with RVFV MP12 for 30 h. Plots present the data as the mean value of 3 biological replicates +/− SEM. Student’s *t*-test: ** *p* < 0.01.

To test this, RIOK3 X2 was overexpressed via transfection of an expression plasmid harboring the open reading frame for RIOK3 X2 in HEK 293 cells, and the cells were subsequently infected with RVFV MP12 at an MOI ~2. RT-qPCR was then used to quantify relative normalized expression of noncanonical NFκB genes CXCL13, CCL19, NIK, and p100. No differences in CXCL13 and CCL19 relative expression were observed, and although expression of NIK was elevated in infected cells transfected with RIOK3 X2 compared to infected cells transfected with an empty vector (EV) control plasmid, the increase was not statistically significant (Figure S6). However, there was a statistically significant elevation in relative expression of p100 in infected cells overexpressing RIOK3 X2, suggesting RIOK3 X2 expression enhances p100 expression and thus likely fuels the noncanonical NFκB pathway (Figure 6a).

Furthermore, to assess whether RIOK3 X2′s engagement of the noncanonical NFκB pathway during RVFV MP12 infection affects the IFN response, relative expression of IFNβ was also measured in MP12-infected HEK 293 cells overexpressing RIOK3 X2. Compared to infected cells transfected with control plasmid, there was a statistically significant decrease in IFNβ expression in infected cells overexpressing RIOK3 X2 (Figure 6b). Again, although it is known that RVFV NSs protein can suppress IFN expression, this experiment examined the effect of exogenous expression of RIOK3 X2 among parallel sets of RVFV-MP12-infected cells. Therefore, we conclude that overexpression of RIOK3 X2 during RVFV MP12 infection elevates p100 expression with a concomitant decrease in IFNβ expression.

Lastly, to directly determine whether the effects of RIOK3 X2 on innate immune responses could be beneficial for RVFV propagation in cell culture, virus titers in supernatants of MP12-infected HEK 293 cells transfected with RIOK3 X2 or MP12-infected HEK 293 cells transfected with control plasmids were quantified by TCID50 assays. TCID50/mL was ~2 logs higher in supernatant from infected cells overexpressing RIOK3 X2 compared to that of controls (Figure 6c). Therefore, expression of RIOK3 X2 during RVFV MP12 infection could be beneficial for the virus, and this might be due, at least in part, to RIOK3 X2 engaging the noncanonical NFκB pathway through supplementing the p100 pool in the infected cell and thereby diminishing the cell’s type 1 IFN response.

## 4. Discussion

A hallmark of the noncanonical NFκB pathway is the appearance of p52, the proteolytically processed subunit of p100. Cellular expression of p52 is exquisitely under tight regulation by the cell, and even small changes in its steady-state levels are associated with activation or suppression of the noncanonical pathway [47,78]. When complexed with RelB, p52 plays a large role in disrupting the type 1 IFN response by competing with the canonical NFκB dimer for the κB binding site of the IFNβ promoter [38]. Here, we show alternative splicing of RIOK3 coincides with enhanced expression of p52′s precursor, p100. Notably, alternative splicing of RIOK3 occurs concurrently with activation of the noncanonical NFκB pathway, and although it is possible that alternative splicing of RIOK3 may be initiated by activation or inhibition of a pathway associated with both TNFα and LIGHT treatments, results here demonstrate alternative splicing of RIOK3 is likely associated with the noncanonical NFκB pathway. Moreover, since overexpression of RIOK3 X2 results in decreased IFNβ expression and increased p100 expression, these results are also consistent with previous results [38] showing that the noncanonical NFκB pathway has an inhibitory effect on the IFN pathway. The link between the NFκB noncanonical pathway and IFN restriction is further emphasized by our finding that increased p100 protein during RVFV MP12 infection is processed to p52.

Since the noncanonical NFκB pathway has an inhibitory effect on the antiviral IFN response, exploiting and co-opting this pathway was proposed to be advantageous for some viruses [34,36,37]. Our results indicate that activation of the noncanonical NFκB pathway occurs early and *before* activation of the canonical NFκB pathway during RFVF MP12 infection, which would not be expected if the activation of the noncanonical pathway we observe was simply part of a coordinated innate immune response to the virus. That is, in a typical cellular innate immune response to viral infection, activation of noncanonical NFκB pathway would be expected to occur significantly later than activation of the canonical pathway. Furthermore, elevated infectious particles observed in RIOK3 X2 overexpressing cells that were shown to have elevated p100 expression further suggest the supportive role this pathway could have in RVFV replication and spread.

Here, we add RVFV to the short list of viruses that use this unusual strategy to enhance their success. Furthermore, we demonstrate that alternative splicing of key genes is instrumental in co-opting the noncanonical NFκB pathway during RVFV infection. Exactly how alternative splicing of RIOK3 is involved in the cell’s activation of the noncanonical NFκB pathway and how RIOK3 X2 expression results in increased expression of p100 will need to be addressed in future studies. However, one potential mechanism for how RIOK3 X2 might be able to increase p100 expression could be through RIOK3 X2 mediating crosstalk between the canonical and noncanonical pathways. Full-length RIOK3 has been shown to inhibit the canonical NFκB pathway [85,86], while expression of RIOK3 X2 correlates with stimulation of this pathway [73]. Therefore, the increased expression of p100 observed here with overexpression of RIOK3 X2 could be a result of activating the canonical NFκB p50:RelA dimer to enter the nucleus to stimulate transcription of p100, which is needed for continued processing of p100 into p52. This would be supported by the finding that p100 expression depends on p50:RelA [65].

It is still unclear whether alternative splicing of RIOK3 alone can activate the noncanonical NFκB pathway or whether RIOK3 alternative splicing occurs downstream of the initial activation of this pathway. Here, we observed alternative splicing of RIOK3 after p52 begins to appear at 24 h post-TNFα treatment, so it is possible that alternative splicing of RIOK3 occurs after the initial activation of the noncanonical pathway. Moreover, alternative splicing in general has been shown to be important for regulating both canonical and noncanonical NFκB pathways [87,88], and intriguingly, it has also been found to be linked to dysregulation of NFκB-mediated immune responses during viral infection [89]. Therefore, further understanding of the onset of alternative splicing of RIOK3 during activation of the noncanonical NFκB pathway could be important for both understanding how innate immune responses are regulated and how they can be dysregulated during viral infection and other disease states.

**Figure 7:**
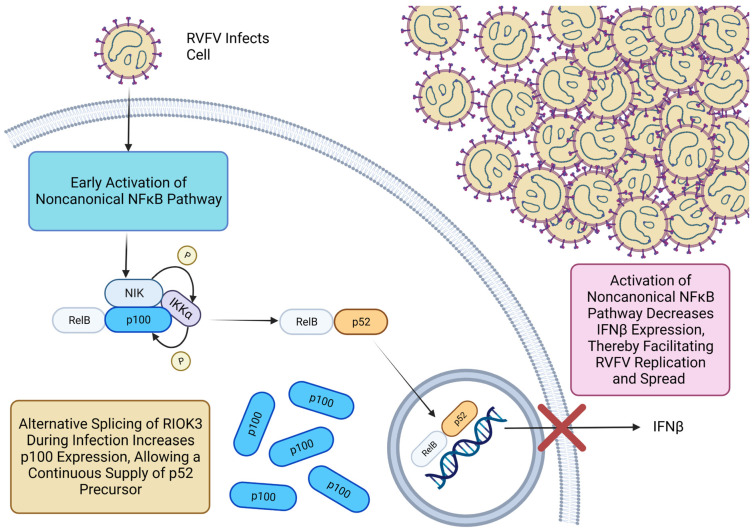
Alternative splicing of RIOK3 replenishes the p100 pool in the cell during RVFV MP12 infection, allowing continuous processing of p100 to p52 during infection.

The present findings help illuminate how cellular innate immune pathways can be targeted and co-opted for viral success (Figure 7). We show the noncanonical NFκB pathway is one innate immune pathway in particular that likely can be exploited by viruses such as RVFV to diminish the potent antiviral IFN response. Notably, alternative splicing of RIOK3, and likely other mRNAs, is an important event that facilitates the switch toward the noncanonical NFκB pathway to mitigate the IFN response. A deeper understanding of viral strategies to undermine cellular defenses could lead to new antiviral therapeutic strategies to combat infections in people and livestock.

## Data Availability

Not applicable.

## Supplementary Materials

The following supporting information can be downloaded at: https://www.mdpi.com/article/10.3390/v15071566/s1, Figure S1: Relative normalized expression of (a) CXCL13, (b) CCL19, (c) NIK, and (d) p100 in HEK 293 cells mock infected for 0 to 30 h; Figure S2: Relative normalized expression of (a) CXCL13, (b) CCL19, (c) NIK, and (d) p100 in mock- and RVFV MP12-infected HepG2 cells; Figure S3: Relative p52 protein levels in (a) HEK 293 cells infected with RVFV MP12 for 6 and 24 h, (b) HEK 293 cells infected with RVFV MP12 for 6 and 30 h, (c) HepG2 cells infected with RVFV MP12 for 6 and 24 h, and (d) HepG2 cells infected with RVFV MP12 for 6 and 30 h; Figure S4: Activation of the (a) noncanonical NFκB pathway and (b) canonical NFκB pathway in HepG2 cells throughout RVFV MP12 infection; Figure S5: Alternative splicing of RIOK3 0-24 h TNFα treatment; Figure S6: Relative Normalized Expression of (a) CXCL13, (b) CCL19, and (c) NIK in mock- or RVFV MP12-infected HEK 293 cells transfected with EV or RIOK3 X2; Table S1:Primer sequences; Table S2: Ratios of antibodies per blocking solution used.
