# Supplementary material for: Alternative Splicing of RIOK3 Engages the Noncanonical NFκB Pathway during Rift Valley Fever Virus Infection
Source: Viruses. 2023 Jul 18;15(7):1566. doi: 10.3390/v15071566 (PMC10383813; doi:10.3390/v15071566)
Supplement: Supplementary file 1 [file viruses-15-01566-s001.zip › viruses-2511403-supplementary.pdf]

Table S1. Primer sequences. CXCL13, CCL19, NIK (MAP3K14), p100 (NFKB2), and IκBα (NFKBIA) qPCR primers used were predesigned by Integrated DNA Technologies (IDT). CXCL13 primers spanned exons 2-3; CCL19 primers spanned exons 1-2; NIK/MAP3K14 primers spanned exons 2-4; p100/NFKB2 primers spanned exons 8-10, and IκBα/NFKBIA primers spanned exons 3-4.

**qPCR Primer**	**qPCR Primer Sequence**
F_IFNβ	5′-AAACTCATGAGCAGTCTGCA-3′
R_ IFNβ	5′-AGGAGATCTTCAGTTTCGGAGG-3′
F_GAPDH	5′-GTCTCCTCTGACTTCAACAGCG-3′
R_GAPDH	5′-ACCACCCTGTTGCTGTAGCCAA-3′
**PCR Primer**	**PCR Primer Sequence**
RIOK3 Exon 5	5′-CCGGTTCCCACTCCTAAAAAGGGC-3′
RIOK3 Exon 10	5′-CCAGCATGCCACAGCATGTTATACTCA-3′
TRA2β Exon 1	5′-AGGAAGGTGCAAGAGGTTGG-3′
TRA2β Exon 3	5′-TCCGTGAGCACTTCCACTTC-3′

Table S2. Ratios of antibodies per blocking solution used.

**Antibody**	**Antibody:Blocking Solution**
Anti-p100/p52 (1°)	1:500
Anti-IκBα (1°)	1:1000
Anti-GAPDH (1°)	1:1000
Anti-β-tubulin (1°)	1:500
HRP Goat Anti-Mouse IgG (2°)	1:10000
HRP Goat Anti-Rat IgG (2°)	1:1000

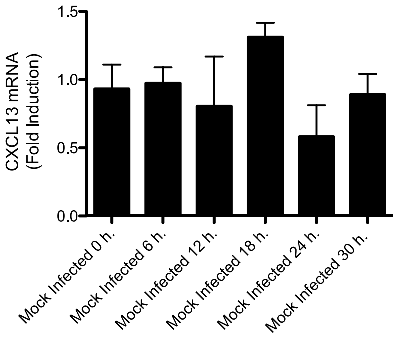

(a)

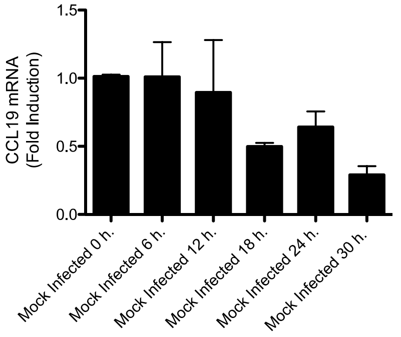

(b)

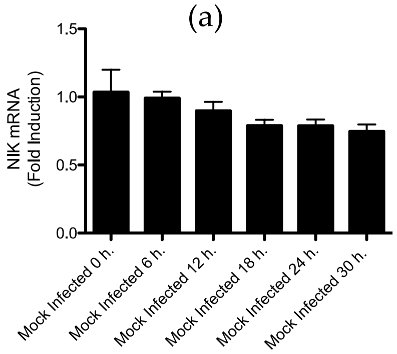

(c)

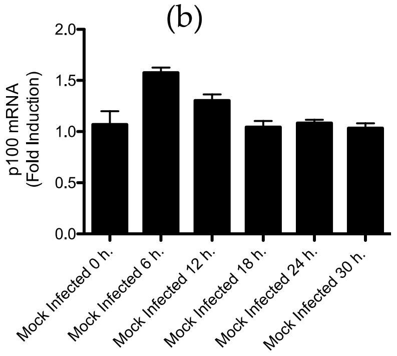

(d)

Figure S1. Relative normalized expression of (a) CXCL13, (b) CCL19, (c) NIK, and (d) p100 in HEK 293 cells mock infected for 0 to 30 h. Quantifications were performed by RT-qPCR, normalized to GAPDH mRNA levels. Graphs present the data as the mean value of 3 biological replicates +/- SEM.

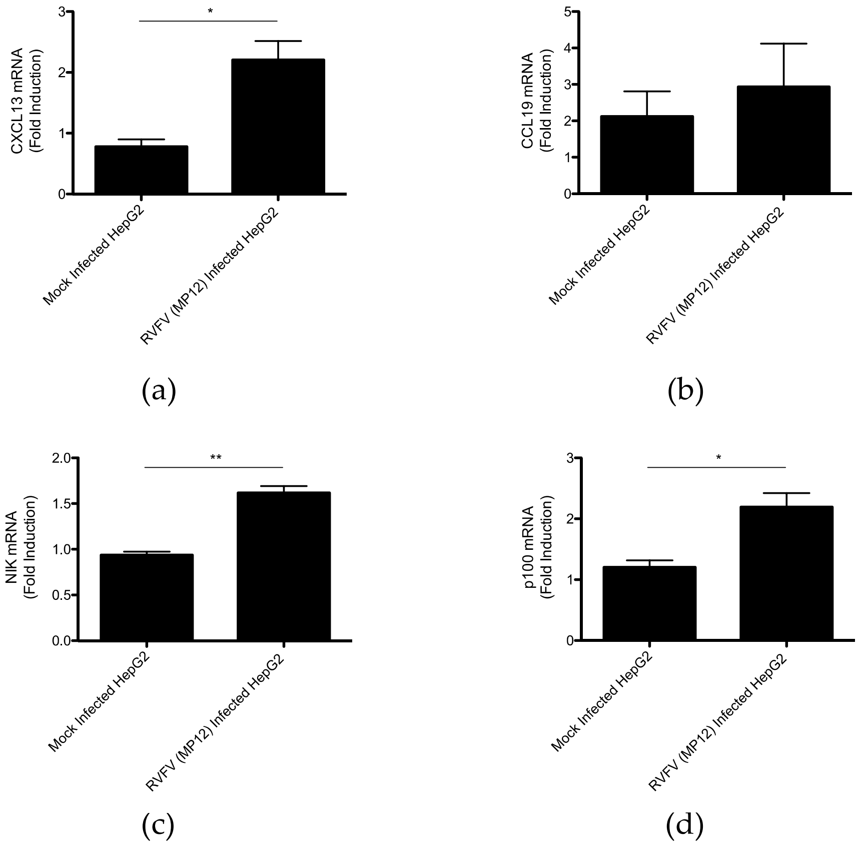

Figure S2. Relative normalized expression of (a) CXCL13, (b) CCL19, (c) NIK, and (d) p100 in mock- and RVFV MP12-infected HepG2 cells. Cells were lysed 24 h.p.i. Quantifications were performed by RT-qPCR, normalized to GAPDH mRNA levels , and graphs present the data as the mean value of 3 biological replicates +/- SEM. Student's *t*-test: * $p < 0.05$, ** $p < 0.01$.

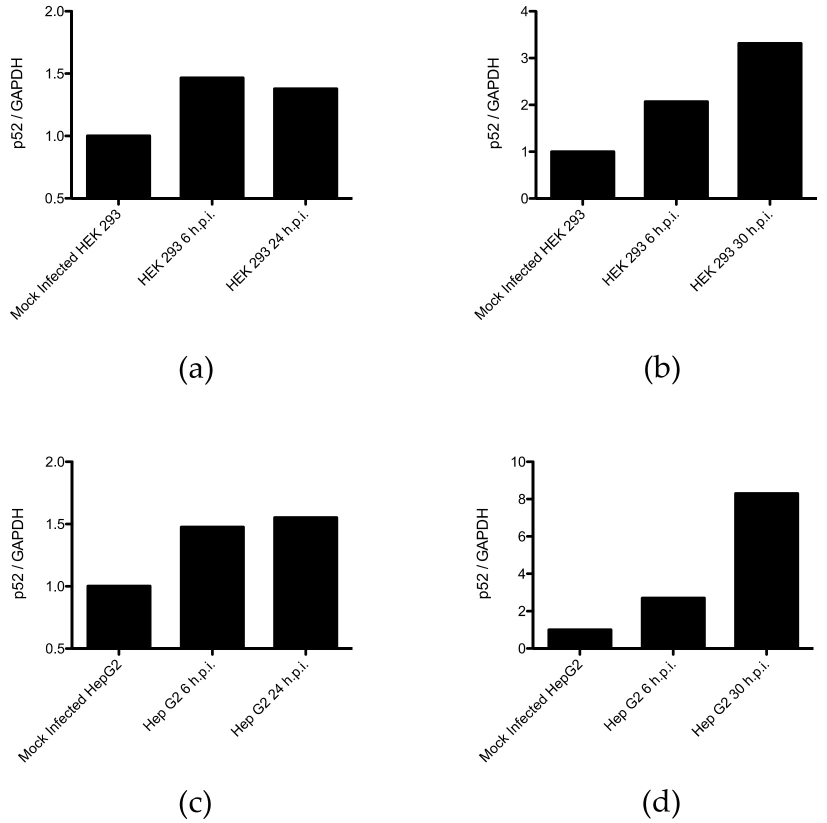

Figure S3. Relative p52 protein levels in (a) HEK 293 cells infected with RVFV MP12 for 6 and 24 h, (b) HEK 293 cells infected with RVFV MP12 for 6 and 30 h, (c) HepG2 cells infected with RVFV MP12 for 6 and 24 h, and (d) HepG2 cells infected with RVFV MP12 for 6 and 30 h. Ratios of band intensities of p52 to GAPDH normalized to ratios in mock infected cells are plotted. Band intensities were quantified from Western blots of representive samples of biological duplicates, and quantifications were performed using ImageJ.

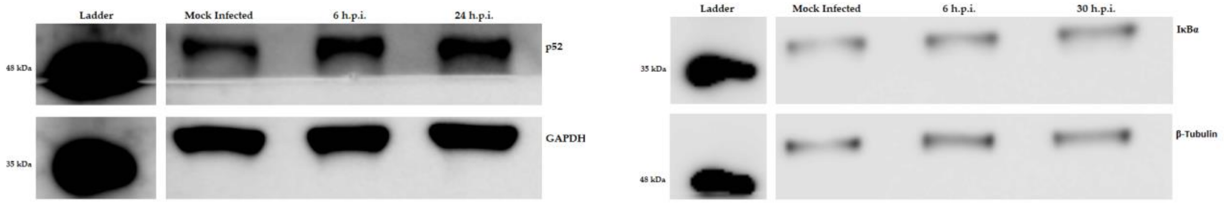

(a) (b)

Figure S4. Activation of the (a) noncanonical NFκB pathway and (b) canonical NFκB pathway in HepG2 cells throughout RVFV MP12 infection. Similar to HEK 293 cells, at 6 h.p.i., accumulation of p52 occurs, but degradation of IκBα does not. In HepG2 cells, degradation of IκBα was not observed at the selected timepoints.

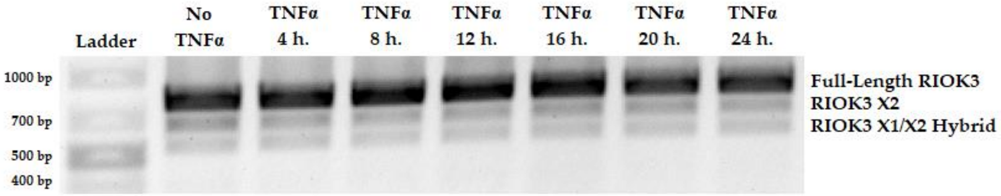

Figure S5. Alternative splicing of RIOK3 0-24 h. TNFα treatment. HEK 293 cells were treated with ~20 ng/mL TNFα.

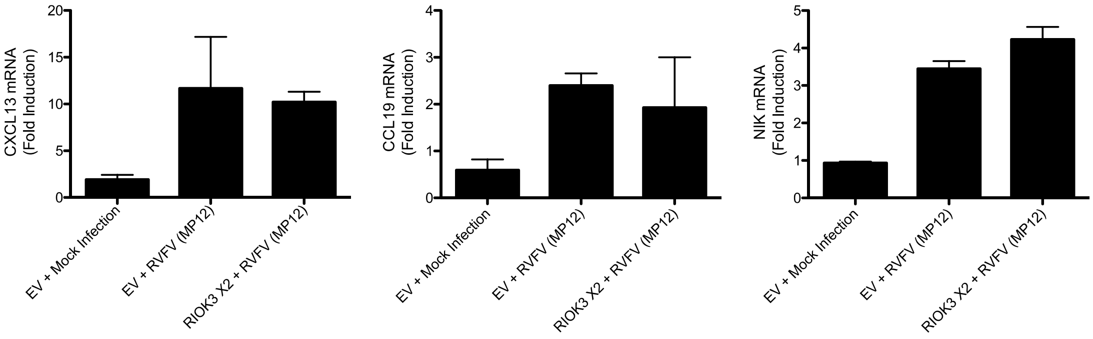

(a) (b) (c)

Figure S6. Relative Normalized Expression of (a) CXCL13, (b) CCL19, and (c) NIK in mock- or RVFV MP12-infected HEK 293 cells transfected with EV or RIOK3 X2. Quantifications were performed by RT-qPCR, normalized to GAPDH mRNA levels , and graphs present the data as the mean value of 3 biological replicates +/- SEM.
